# A Nomogram Combining a Four-Gene Biomarker and Clinical Factors for Predicting Survival of Melanoma

**DOI:** 10.3389/fonc.2021.593587

**Published:** 2021-04-01

**Authors:** Chuan Zhang, Dan Dang, Yuqian Wang, Xianling Cong

**Affiliations:** ^1^ Department of Pediatric Surgery, The First Hospital of Jilin University, Changchun, China; ^2^ Department of Neonatology, The First Hospital of Jilin University, Changchun, China; ^3^ Scientific Research Center, China-Japan Union Hospital of Jilin University, Changchun, China; ^4^ Department of Dermatology, China-Japan Union Hospital of Jilin University, Changchun, China

**Keywords:** prognostic biomarker, nomogram, microenvironment, melanoma, immune genes

## Abstract

**Background:**

Currently there is no effective prognostic indicator for melanoma, the deadliest skin cancer. Thus, we aimed to develop and validate a nomogram predictive model for predicting survival of melanoma.

**Methods:**

Four hundred forty-nine melanoma cases with RNA sequencing (RNA-seq) data from TCGA were randomly divided into the training set I (n = 224) and validation set I (n = 225), 210 melanoma cases with RNA-seq data from Lund cohort of Lund University (available in GSE65904) were used as an external test set. The prognostic gene biomarker was developed and validated based on the above three sets. The developed gene biomarker combined with clinical characteristics was used as variables to develop and validate a nomogram predictive model based on 379 patients with complete clinical data from TCGA (Among 470 cases, 91 cases with missing clinical data were excluded from the study), which were randomly divided into the training set II (n = 189) and validation set II (n = 190). Area under the curve (AUC), concordance index (C-index), calibration curve, and Kaplan-Meier estimate were used to assess predictive performance of the nomogram model.

**Results:**

Four genes, i.e., *CLEC7A*, *CLEC10A*, *HAPLN3*, and *HCP5* comprise an immune-related prognostic biomarker. The predictive performance of the biomarker was validated using tROC and log-rank test in the training set I (n = 224, 5-year AUC of 0.683), validation set I (n = 225, 5-year AUC of 0.644), and test set I (n = 210, 5-year AUC of 0.645). The biomarker was also significantly associated with improved survival in the training set (*P* < 0.01), validation set (*P* < 0.05), and test set (*P* < 0.001), respectively. In addition, a nomogram combing the four-gene biomarker and six clinical factors for predicting survival in melanoma was developed in the training set II (n = 189), and validated in the validation set II (n = 190), with a concordance index of 0.736 ± 0.041 and an AUC of 0.832 ± 0.071.

**Conclusion:**

We developed and validated a nomogram predictive model combining a four-gene biomarker and six clinical factors for melanoma patients, which could facilitate risk stratification and treatment planning.

## Introduction

Cutaneous melanoma is the deadliest type of skin cancer (1, 2), and its morbidity has been on the rise annually, especially in the Caucasian population (3, 4). As melanoma is generally recognized as a highly heterogeneous cancer (5) and immunotherapy remains the preferred treatment for advanced melanoma (6), immune-related biomarkers have been exploited as prognostic signatures of melanoma (7–10). However, the current existing immune-related prognostic biomarkers have their limitations. For instance, some biomarkers contain a relatively large number of genes that reduces their potential applicability to some extent (7, 8), while for others, there is a lack of detailed information regarding the potential mechanism and clinical relevance (8, 9). Therefore, the identification of a comparatively reliable and applicable prognostic biomarker for melanoma in order to guide clinical decision-making is essential.

Considering the advancements in gene sequencing technology, a set of gene databases, such as The Cancer Genome Atlas (TCGA) (11, 12) and Gene Expression Omnibus (GEO) have emerged as popular guide sources. A series of bioinformatics tools, including weighted gene co-expression network analysis (WGCNA) (13), cell-type identification by estimating relative subsets of RNA transcripts (CYBERSORT) (14), gene set enrichment analysis (GSEA) (15, 16), and least absolute shrinkage and selection operator (LASSO), have been used to process such big data. The strategy of using a combination of these databases and bioinformatics tools in scientific practice is supported by the reliability of such approaches (17–21).

To identify an immune-related prognostic biomarker and develop a new nomogram predictive model for melanoma patients, we analyzed the RNA sequencing (RNAseq) data and the corresponding clinical data from TCGA and GEO databases using bioinformatic tools. The findings would show useful prognostic factors and a nomogram for predicting survival in melanoma patients. Researchers, clinicians, and patients would handily forecast the survival probability for each individual patient using this nomogram.

## Materials and Methods

### Data Acquisition

Four hundred seventy-two melanoma cases with RNA sequencing data were download from TCGA, and 449 of them with complete survival data were randomly divided into the training set I (n = 224) and validation set I (n = 225) (**Table S1**). Two hundred fourteen melanoma cases with RNA sequencing data and survival data were obtained from Lund cohort of Lund University (available in GSE65904) (22, 23) and 210 of them with complete survival data were utilized as an external test set (**Table S1**). The above three sets were used to identify and validate a prognostic gene biomarker.

Four hundred seventy melanoma cases with clinical data were obtained from TCGA and 91 cases with missing clinical data were excluded from the study. Of them, 379 met our inclusion criterion that they do not contain any missing data for selected variables including age, gender, overall survival time, survival status, and clinical stage. The 379 cases were subsequently randomly assigned to the training set II (n = 189) and validation set II (n = 190) (**Table S2**), which were used to develop and validate a nomogram predictive model. In developing the nomogram, the four-gene biomarker and clinical characteristics were used as variables.

Immune, stromal, and estimate scores of each patient were available from the ESTIMATE database (**Table S2**) (24). A total of 5,559 human immune genes were downloaded from the InnateDB database (**Table S2**) (25).

### WGCNA

WGCNA, a reliable and approved bioinformatics method, was employed to identify immune-related modules. We first removed outlier genes and genes expressed at extremely low levels from the data. Construction of a weighted gene network involves the choice of the soft thresholding power *β* to which co-expression similarity is raised to calculate adjacency. Based on the criterion of approximate scale-free topology, we chose 14 as the soft threshold. Using the soft threshold, we calculated the adjacency (co-expression similarity) and generated a hierarchical clustering tree. The dynamic tree cut could enable the identification of modules with very similar expression profiles. The modules with highly co-expressed genes were merged. Finally, we correlated modules with external traits (herein i.e. immune score) and identified the most relevant module.

### Database for Annotation, Visualization, and Integrated Discovery (DAVID) Online Tool

DAVID (version 6.8) (26) is an online bioinformatics tool that provides a comprehensive set of functional annotation tools for interpreting the biological meaning underlying specific gene sets. Herein, DAVID was used to perform the Kyoto Encyclopedia of Genes and Genomes (KEGG) pathway and Gene Ontology (GO) analysis based on the genes from the most relevant module identified using WGCNA. GO analysis can provide information on functions of genes. KEGG pathway analysis can suggest the possible involved signaling pathways of a gene set. The brief operation process is as follows. Briefly, the symbols of the genes to be analyzed were uploaded onto the website, and *Homo sapiens* is selected as the species. Next, GO-BP-DIRECT, GO-CC-DIRECT, GO-MF-DIRECT, and KEGG PATHWAY were selected to perform functional annotation. All other parameters were set as default.

### Identification and Validation of the Immune-Gene Biomarker

Overlapping genes from the most relevant module from WGCNA, immune genes from the IRIS database, and genes from GSE65904, were then analyzed using the univariate Cox regression analysis and least absolute shrinkage and selection operator (LASSO) regression analysis based on the training set I. The genes obtained from the above analysis were used for developing a prognostic immune-gene biomarker using the multivariate Cox regression analysis based on the training set I.

The prognostic signature can be quantified by calculating risk scores using multivariate Cox regression model. The predictive performance of the immune gene biomarker was assessed using area under the curve (AUC), calibration curve, Kaplan-Meier estimate in the training set I, validation set I, and test set, respectively.

### Differentially Expressed Tumor-Infiltrating Immune Cell (TIICs) Analysis

TIICs between different groups were compared based on their abundance in each melanoma sample, which was calculated using CYBERSORT (**Table S2**). CYBERSORT is an *in silico* algorithm that enables the precise estimation of immune cell fractions based on RNAseq profiles of bulk samples (14). The accuracy of CYBERSORT has been demonstrated by immunohistochemistry and flow cytometry. Statistical parameters used include: *p*-value (*t*-test) and log_2_ fold change (logFC).

### GSEA

GSEA was performed using the GSEA software (version 3.0) to detect any differences in the KEGG pathways between the low-risk and high-risk groups. The operating parameters were set as follows: the number of permutations at 1,000, weighted enrichment statistic, metric for ranking genes (Signal2Noise), max size (500), and min size (15).

### Nomogram Development and Validation

To investigate the prognostic significance of the immune-gene biomarker in combination with common clinical characteristics, we planned to develop a predictive nomogram combining the immune-gene biomarker and clinical factors for melanoma patients. First, univariate Cox regression was used to screen for clinical characteristics that were significantly correlated with overall survival in the training set II. Second, clinical characteristics with a *P* value less than 0.05 were used to developed a nomogram using multivariate Cox regression model.

To validate the proposed nomogram, four criteria were utilized to assess prediction performance in the validation set II. First, the cases were grouped according to their predicted risk score, and Kaplan-Meier survival curves and log rank test were used to compare survival differences among the groups. Second, a concordance index (C-index) was calculated to estimate the similarity between the ranking of true survival time and of predicted risk score. The theoretical value of the C-index is between 0 and 1; a C-index larger than 0.5 indicates prediction performance better than random guessing. Third, integrated AUC was calculated. Fourth, calibration curves were plotted to evaluate the consistency between predicted survival probability and actual survival proportion at 4 years. A perfect prediction would result in a 45-degree calibration curve (i.e. the identity line).

### Statistical Analyses

All statistical analysis was performed in the R Studio software (version 3.6.1). R packages “WGCNA” (13), “Vennerable” (27), “glmnet” (28, 29), “ggplot2” (30), “survival” (31), “survminer” (32), “survivalROC” (33), “rms” (34), “pROC” (35), “forestplot” (36) were used. Continuous values between the two groups were analyzed using *t*-tests. Non-parametric comparison between the two groups was performed using the Wilcoxon test. *P* < 0.05 was considered statistically significant.

## Results

### Study Protocol

**Figure 1:**
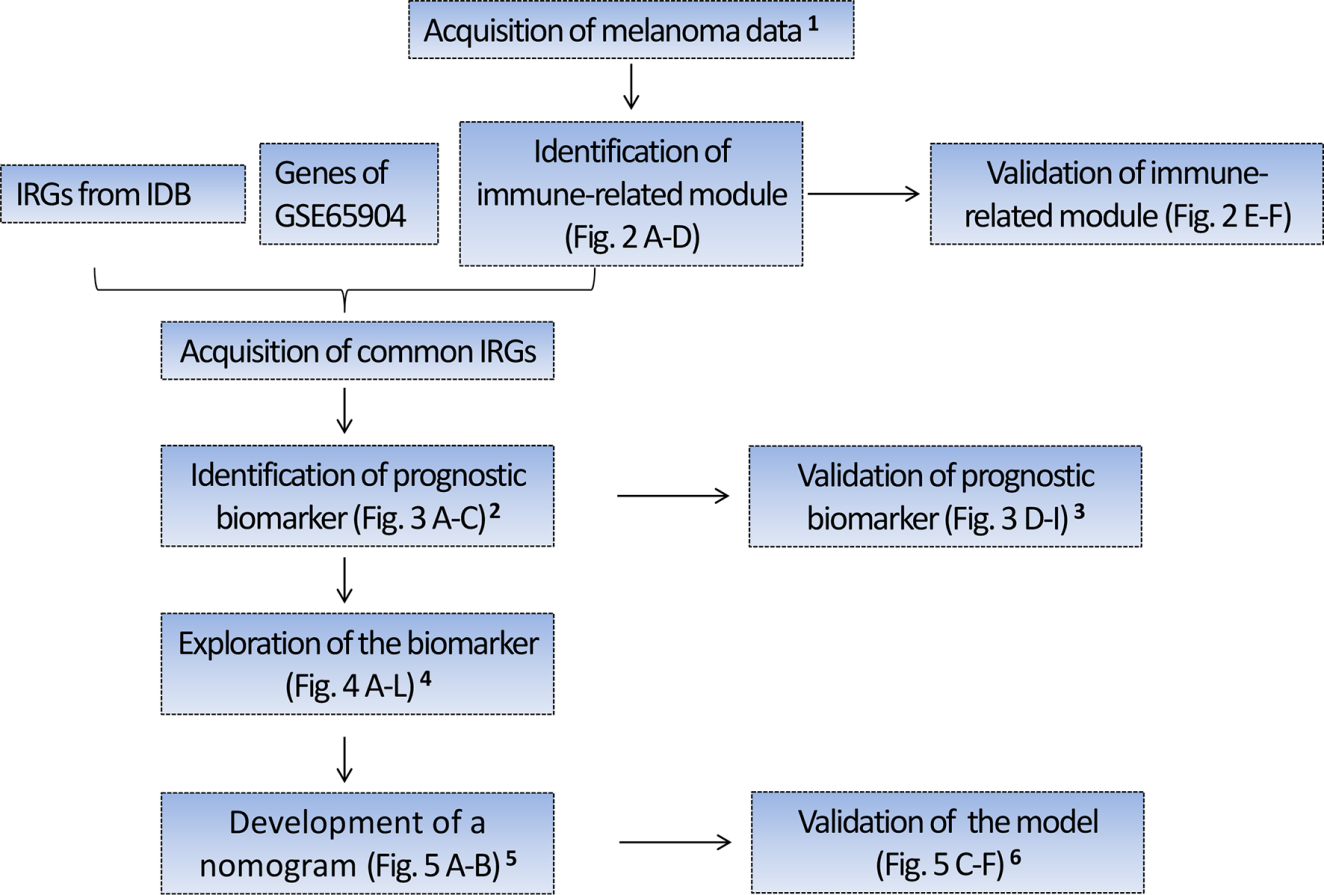
Flow chart depicting the protocol. ^1^ Four hundred forty-nine melanoma cases with RNA sequencing (RNA-seq) data from TCGA were randomly divided into the training set I (n = 224) and validation set I (n = 225), 210 melanoma cases with RNA-seq data from Lund cohort of Lund University (available in GSE65904) were used as an external test set. The above three sets were used to identify and validate a prognostic gene biomarker. ^2^ Based on the training set I, we identified a four-gene biomarker from 56 IRGs using the univariate Cox regression analysis and LASSO regression analysis. ^3^ The predictive performance of the four-gene biomarker was validated in the training set I, validation set I, and an external test set (GSE65904). ^4^ Exploration of the biomarker includes the association of the four-gene biomarker with the patient’s survival, immune score, clinical stage, tissue pathological type, tumor-infiltrating immune cells, and KEGG pathway. ^5^ Four hundred seventy melanoma cases with clinical data were obtained from TCGA and 91 cases with missing clinical data were excluded from the study. Three hundred seventy-nine cases with complete clinical data were subsequently randomly assigned to the training group II (n = 189) and validation group II (n = 190), which were used to develop and validate the nomogram predictive model. In developing the nomogram, the four-gene biomarker and clinical characteristics were used as variables. ^6^ The predictive power of nomogram combining the four-gene biomarker and clinical characteristics was assessed in the training set II and validation set II. IRGs, immune-related genes; IDB, the InnateDB database.

The schematic diagram of the study protocol is shown in **Figure 1**.

### Identification and Validation of the Immune-Related Module

**Figure 2:**
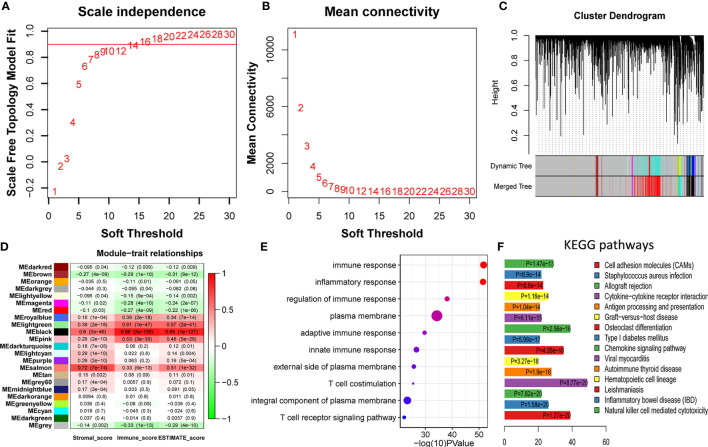
Identification and validation of immune-related modules **(A, B)** Analysis of network topology for various soft-thresholding powers. **(A)** This panel shows the scale-free fit index as a function of the soft-thresholding power. **(B)** This panel displays the mean connectivity as a function of the soft-thresholding power. **(C)** Clustering dendrogram of genes, with dissimilarity based on topological overlap, together with assigned merged module colors and the original module colors. Each color represents a module. **(D)** Module-trait association. Each row represents a module, and each column represents a trait. Each cell contains the corresponding correlation and *P* value. Module Black (MEblack) is the most immune score-related (*P* = 3e-155, *R* = 0.89). **(E)** The top 10 categories are all immune-related in GO enrichment analysis based on genes in Module Black, supporting genes in Module Black are indeed immune-related. **(F)** Most of the top 15 KEGG pathways are also immune-related, underscoring genes of Module Black are related to immunity.

To identify the immune-related module, the melanoma RNAseq data and the corresponding immune scores for each patient were analyzed using WGCNA. Module Black comprising 809 genes was identified as the strongest immune-related module. From 53,898 genes, we filtered out the outlier genes and genes expressed at extremely low levels to obtain 21,194 candidates for WGCNA. The soft threshold was determined as 14 (**Figures 2A, B**). All the genes were classified into 27 modules. After merging the highly co-expressed modules, 23 modules were eventually obtained (**Figure 2C**). Among them, Module Black was the strongest immune-related module (*P* = 3e-155, *R* = 0.89). Intriguingly, Module Black was also significantly associated with stromal and estimate scores in melanoma (**Figure 2D**).

To verify whether the selected module correlated with immunity, 809 genes in Module Black were analyzed using DAVID. As shown in **Figures 2 (E, F)**, the top five relevant pathways in both GO analysis and KEGG pathway were found to be immune-related.

### Identification and Validation of Prognostic Immune-Gene Biomarker

**Figure 3:**
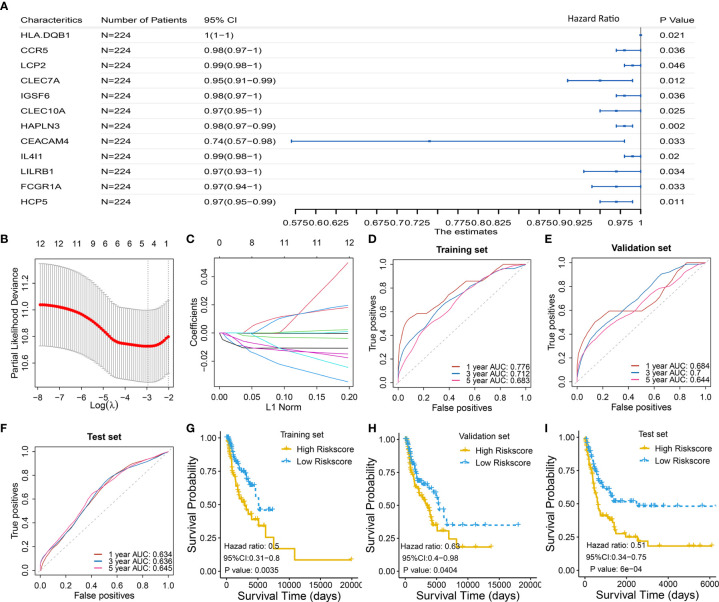
Identification and validation of four-gene biomarker **(A)** Univariate Cox regression were used to screen for genes that were significantly correlated with overall survival in the training set I (n = 224). Twelve genes with *P* value less than 0.05 were significantly associated with overall survival, as shown in the forest plot. **(B–C)** LASSO regression was used to further eliminate redundant genes. The resulting four genes of *CLEC7A*, *CLEC10A*, *HAPLN3*, and *HCP5* were used to develop a four-gene biomarker based on multivariate Cox regression model. **(B)** Tuning parameter (λ) selection in the LASSO model used 10-fold cross-validation *via* minimum criteria. AUC was plotted *versus* log(λ). **(C)** Coefficient profiles of the fractions of 12 immune-related genes. **(D–F)** One-, 3-, and 5-year AUC were calculated for the prognostic four-gene biomarker, showing good predictive performance in the training set I, validation set I, and test set. **(G–I)** Risk scores of melanoma cases were calculated according to multivariate Cox regression model of the four genes, and grouped into low-risk and high-risk group using median risk score as threshold. Low-risk group has a significant longer survival compared to high-risk group, in the training set I, validation set I, and test set.

To select the qualified immune genes for developing an immune-gene biomarker, 56 overlapping genes were obtained by intersecting the 809 genes from Module Black (**Table S2**), 5,559 immune genes from the IRIS database, and 2,786 genes from GSE65904 (**Table S1**). First, we randomly divided 449 melanoma patients with RNA sequencing data and complete survival data from TCGA cohort into the training set I (n = 224) and validation set I (n = 225) (**Table S1**). The selected 56 genes were analyzed as variables using the univariate Cox regression analysis based on the training set I. Twelve genes with a *P* value less than 0.05 (*HLA-DQB1*, *CCR5*, *LCP2*, *CLEC7A*, *IGSF6*, *CLEC10A*, *HAPLN3*, *CEACAM4*, *IL4I1*, *LILRB1*, *FCGR1A*, and *HCP5*) were selected in the univariate Cox regression model (**Figure 3A**).

Then, the 12 genes were further screened using LASSO regression model, and four gene of *CLEC7A*, *CLEC10A*, *HAPLN3*, and *HCP5* were eventually selected to develop a four-gene biomarker using the multivariate Cox regression model (**Figures 3B, C**; detailed computational process is available in **Table S3**).

The proposed four-gene biomarker was validated in the validation set I (n = 225) and an independent testing set (GSE65904, n = 210). The 5-year AUC of the four-gene biomarker was 0.683, 0.644 and 0.645 in the training set, validation set and external test set, respectively (**Figures 3D–F**). Next, we calculated risk score of each patient using the multivariate Cox regression model based on the four-gene biomarker. The survival difference between two groups, which were grouped by the median predicted risk score, was significant (*P* value < 0.05; **Figures 3G–I**).

### Relationship of Four-Gene Biomarker With Clinical Factors

**Figure 4:**
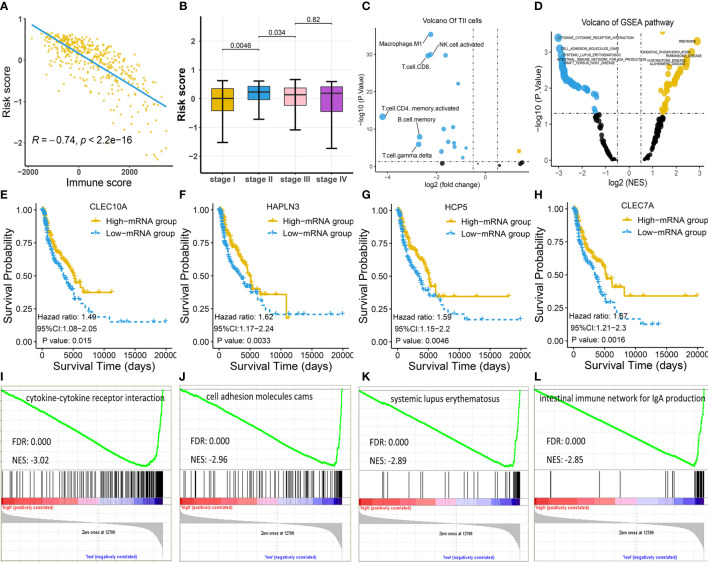
Exploration of the four-gene biomarker **(A–C)** Risk scores of melanoma patients from TCGA cohort were calculated according to the four-gene biomarker, and the association of risk scores with common clinical characteristics were investigated. **(A)** Risk score was negatively correlated with immune score, consistent with low-risk patients who had a prolonged survival in melanoma. **(B)** There was a marked difference in risk score between stage I and stage II, as well as between stage II and stage III, implying qualitative change occurred after stage II. **(C)** The numbers of M1 macrophage, NK, CD4^+^, and CD8^+^ T cells were critically elevated in the low-risk group (the blue dot indicates TIICs whose counts are increased in the low-risk group, while the yellow dot indicates TIICs whose counts are increased in the high-risk group). **(D)** KEGG pathway analysis by GSEA displayed significantly differentially enriched pathways between the low-risk and high-risk groups. Each blue dot represents a significantly enriched pathway in the low-risk group, while yellow dot represents that in the high-risk group. **(E–H)** Four genes, *CLEC7A*, *CLEC10A*, *HAPLN3*, and *HCP5*, had a significant relevance with respect to survival; this is indicative of their anti-tumoral roles in melanoma. **(I–L)** The top four pathways in the low-risk group were all immune-related, indicating more active immune function in low-risk group compared to in the high-risk group.

Since the identified four-gene biomarker manifested prognostic relevance in patients with melanoma, we wondered if four-gene biomarker was significantly correlated with other clinical factors in melanoma. We found that risk score was negatively associated with immune score in melanoma (*P* < 0.05), indicating that the low-risk score was potentially attributed to activated immune function (**Figure 4A**). In addition, we analyzed the relationship between risk score and clinical stages, revealing that there was a significant difference in the risk score between stage I and stage II, as well as between stage II and stage III (**Figure 4B**). The four genes of *CLEC7A*, *CLEC10A*, *HAPLN3*, and *HCP5* were analyzed using log-rank tests with TCGA data, and they all showed significant survival significance (*P* < 0.05; **Figures 4E–H**), implying their protective effects on melanoma.

To explore the association between risk score and TIICs, we divided the TCGA cohort into the low-risk and high-risk groups based on the median risk score and conducted a differentially expressed TIIC analysis between these groups based on the TIIC abundance in each sample. TIIC abundance in each tissue was calculated using CYBERSORT, and the results are shown in **Supplementary Table S3**. The findings revealed that M1 macrophage, CD4^+^ T cells, CD8^+^ T cells, and natural killer (NK) cells were the top four upregulated TIICs in the low-risk group (**Figure 4C**), further underscoring activated immune function in this group.

Consistent with the above findings, GSEA also revealed that the top four KEGG pathways in the low-risk group were all immune-related [cytokine-cytokine receptor interaction; cell adhesion molecules (CAMs); systemic lupus erythematosus; the intestinal immune network for IgA production], while the top four pathways in the high-risk group were not immune-related (**Figures 4D, I–L**; **Table S4**).

### Development and Validation of a Nomogram

**Figure 5:**
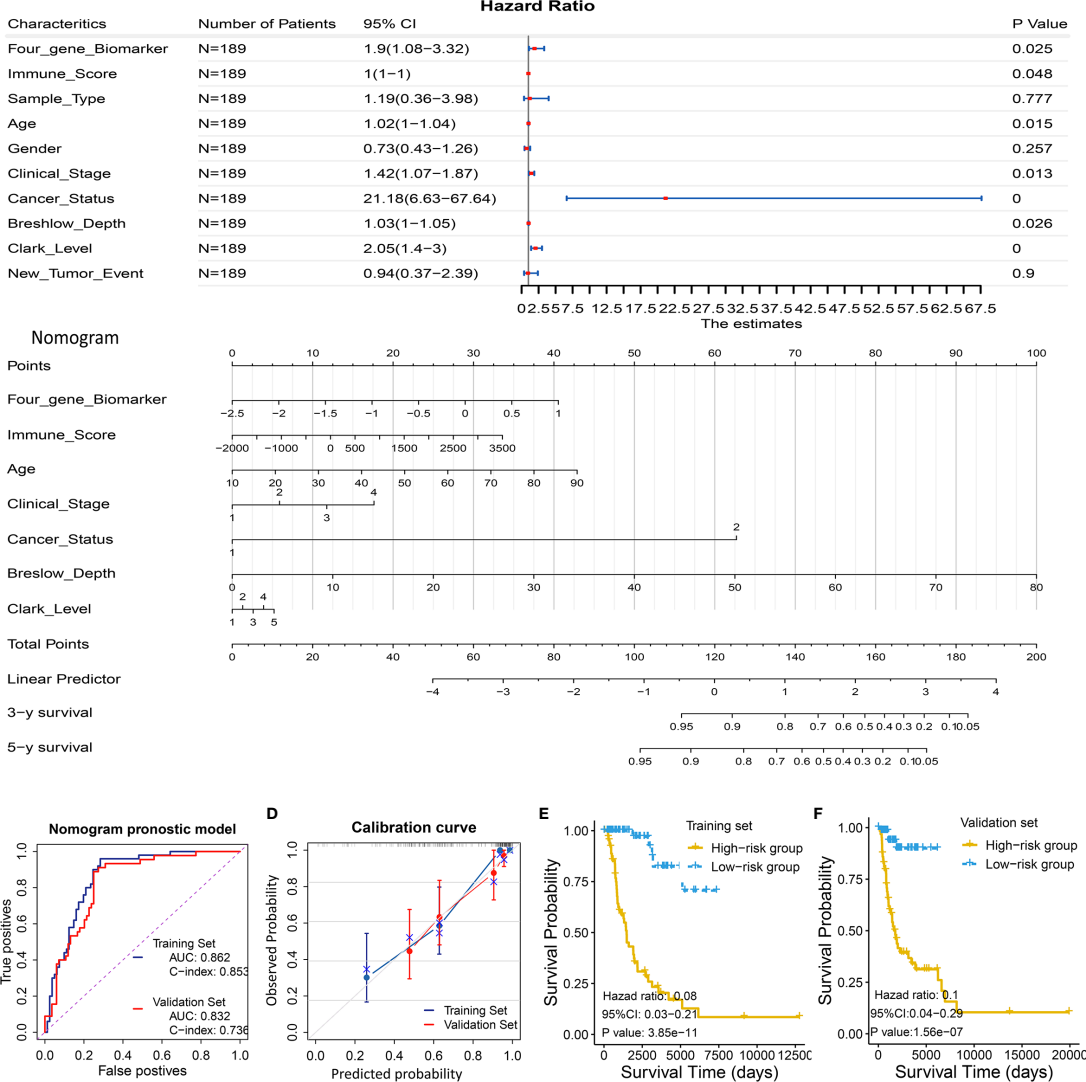
Development and validation of a predictive nomogram for predicting survival probability. **(A, B)** Development of a predictive nomogram combing the four-gene biomarker and clinical factors in melanoma based on the training set II. **(A)** Univariate Cox regression were used to screen for clinical factors that were significantly correlated with overall survival in the training set II (n = 189), as shown in the forest plot. Seven factors including the four-gene biomarker were significantly associated with overall survival. **(B)** A nomogram combing the four-gene biomarker and clinical factors for predicting 3- and 5-year overall survival for melanoma patients. Cancer status represents personal cancer status (with tumor/tumor-free), which is one of the clinical characteristics for melanoma patients. **(C–F)** Four criteria were utilized to assess the predictive performance in the training set II and validation set II. **(C)** AUC and C-index were calculated for the nomogram prognostic models in the training set (n = 189) and validation set (n = 190). AUC of the nomogram was 0.862 ± 0.062 and 0.832 ± 0.071, and C-index was 0.853 ± 0.024 and 0.736 ± 0.041, in the training group and validation group, respectively. **(D)** Calibration curves of nomograms in training set and validation set. X-axis represents predicted probability and Y-axis represents true probability. Each point in the plot represents a subgroup of patients. Error bars represent 95% confidence intervals. 45° represents perfect prediction, and the actual performances of our nomogram are very well. **(E, F)** The resulting nomogram prognostic model was utilized to calculate risk score of cases in the training set and validation set. Low risk score subgroup had a significantly improved survival compared to high risk score (grouped according to median risk score value), in training set and validation set. The findings support the predictive power of the proposed nomogram.

Considering the prognostic significance of the four-gene biomarker, we sought to combine it with nine common clinical factors to better predict survival of melanoma patients. We first conducted a univariate Cox regression analysis to examine the prognostic significance of the four-gene biomarker and nine clinical factors, including age, gender, clinical stage, breshlow depth, clark level, tissue sample type (primary or metastatic melanoma), cancer status (with tumor or tumor-free), immune score, and new tumor event, based on the training set II. Personal cancer status (with tumor/tumor-free) is one of the clinical characteristics for melanoma cases from the TCGA cohort (**Figure 5A**). Herein, we defined the personal cancer status of the tumor as cancer status. The results showed that seven factors could be used as effective prognostic characteristics for melanoma, including four-gene biomarker, immune score, age, clinical stage, cancer status, breslow depth, and clark level. Thus, the seven factors were used to developed a nomogram prognostic model based on the training set II (n = 189) (**Figure 5B**).

The proposed nomogram was assessed in the validation set II (n = 190), with a C-index of 0.736 ± 0.041 and an AUC of 0.832 ± 0.071 (**Figure 5C**). A calibration curve at 4 year (**Figure 5D**) also showed high consistency between predicted survival probability and actual survival proportion. The survival difference between two groups, which were grouped by the median predicted risk score, was significant (*P* < 0.05; **Figures 5E, F**). The predicted risk score was calculated by adding up the score of each item using the nomogram depicted in **Figure 5B**.

## Discussion

We developed and validated a four-gene biomarker and a nomogram prognostic model combining a four-gene biomarker and six clinical factors for melanoma patients. The four genes (*CLEC7A*, *CLEC10A*, *HAPLN3*, and *HCP5*) were also identified as meaningful anti-tumoral genes in melanoma. Among these, *CLEC7A*, *CLEC10A*, and *HAPLN3* have not yet been reported to be correlated with melanoma before. Furthermore, we revealed that the counts of several TIILs (M1 macrophage, NK cells, CD4+ T cells, and CD8+ T cells) were significantly elevated in the low-risk populations. In addition, the number of activated immune pathways was higher in the low-risk populations than that in the high-risk counterparts, which could provide insights for future studies. All these findings may contribute to the development of novel strategies for melanoma treatment and may provide an opportunity to perform in-depth research into the immune underpinning of melanoma.

One of the main findings of this study is the optimized immune-related prognostic biomarker comprising four immune genes (*CLEC7A*, *CLEC10A*, *HAPLN3*, and *HCP5*). The existing prognostic biomarkers usually contained at least 10 genes (37–41), a feature that would substantially reduce their clinical applicability. In contrast, the prognostic immune-related biomarker identified herein comprises only four genes, and is therefore more convenient for clinical application. Meanwhile, the established model herein had been validated in three subsets, i.e., training, validation, and external tests, further supporting its extensive applicability.

Three genes, i.e., *CLEC7A*, *CLEC10A*, and *HAPLN3* have been reported to exhibit prognostic significance with respect to melanoma for the first time, while *HCP5* has been reported to inhibit the development of cutaneous melanoma (42). *CLEC7A* (also known as dectin-1) encodes for a pattern-recognition receptor expressed by myeloid phagocytes (macrophages, dendritic cells, and neutrophils) that can directly drive the antimicrobial activity (43, 44). Although *ClEC7A* activation on macrophages has been reported to induce the development of pancreatic cancer and peri-tumoral immune tolerance (45), there is no evidence regarding the relationship between *CLEC7A* and melanoma. We revealed that elevated expression of *CLEC7A* could result in enhanced anti-tumoral immunity and may be correlated with prolonged survival in melanoma. *CLEC7A* could function *via* NK cells and M1 macrophages to suppress metastasis (46), consistent with the increase in the population of NK cells and M1 macrophages in the low-risk group in our study. However, the precise underlying mechanism remains largely unknown. *CLEC10A* was reported to play an important role in immune cell maturation and *CLEC10A* expression is known to correlate with improved survival in breast and ovarian cancers (47–49). Furthermore, *CLEC10A* could suppress HDM-induced Toll-like receptor 4 (TLR4)-mediated inflammatory cytokine production in mice to maintain homeostasis against inflammation. Our results demonstrate that high *CLEC10A* expression was associated with improved melanoma survival and immune scores, indicating its potential role in anti-tumoral immunity. Meanwhile, over-modulated expression of *HAPLN3* was suggested to relate with the initiation of breast cancer (50); however, its function in melanoma is presently unclear. The role of the long non-coding RNA (lncRNA) *HCP5* in cancer remains controversial. Some reports suggest that *HCP5* could induce tumor progression in cases of follicular thyroid carcinoma and lung adenocarcinoma (51–53), while others claim that *HCP5* may suppress the development of cutaneous melanoma by modulating *RARRES3* expression by sponging miR-12 (42). Consistent with previous studies, we observed an association between upregulated *HCP5* expression and improved survival in melanoma and that *HCP5* might boost the anti-tumoral immunity. Nevertheless, further *in vitro* and *in vivo* investigations are warranted to study the role of these four pivotal genes in melanoma and their precise mechanisms of action.

Our study demonstrates that the risk score was significantly associated with the immune score and tumor-infiltrating immune cell abundance, thereby supporting the importance of immune function in melanoma. M1 macrophages are associated with improved prognostic outcomes in melanoma (54–57). NK cells were confirmed to induce macrophage polarization toward the M1 phenotype and suppress tumor growth (58). Similarly, we observed higher immune scores and high abundance of M1 macrophage, CD4+, CD8+, and NK cells in the low-risk population. Further, we revealed the proportion of melanoma-infiltrating immune cells and their association with melanoma prognosis.

Another important finding is the activation of several immune pathways in the low-risk group and its correlation with prolonged survival in melanoma. Cytokine-cytokine receptor interaction pathway was the most significant in the low-risk group; this pathway plays an important role in recovery after infection with the respiratory syncytial virus as well as in colorectal cancer, renal cell carcinoma, and esophageal cancer (59–62).

This study has important implications for the treatment as well as prognosis of melanoma. First, our study provides a new prognostic biomarker and a new nomogram that could aid clinical treatment strategies for melanoma. Second, we revealed several critical immune genes, cells, and pathways that could serve as promising therapeutic targets in melanoma.

This study has a few limitations that warrant further research. First, as the four immune genes exhibit critical significance in melanoma prognoses, further *in vitro* and *in vivo* studies are required to explore their physiological mechanisms of actions. In addition, M1 macrophage, NK, CD8^+^, and CD4^+^ T cells are indicated to benefit the survival of melanoma patients, warranting further investigation regarding the precise underlying mechanism. Third, the performance of the four-gene biomarker and similar prediction models was not statistically compared. To clarify this, we searched the PubMed database and observed that the prognostic power of the established model is still acceptable and stable as compared with that of previously established models. The 5-year AUC values of tROC for the present biomarker in training, validation, and testing sets were 0.683, 0.644, and 0.645, respectively, while those of the other models were 0.723, 0.560, and 0.682 (63); 0.648, 0.544, and 0.755 (64); and 0.68,0.65, and 0.63 (65), respectively. However, the nomogram prognostic model combing clinical factors showed a better prediction power, with an AUC value of 0.862 in the training group and 0.832 in the validation set, respectively. The drawback is that the nomogram model was not assessed in an external test set for lacking a data set with some routinely available clinical data including breslow depth, clark level, clinical stage, and survival information.

In conclusion, we successfully constructed and validated a four-gene biomarker and a nomogram prognostic model by investigating data from TCGA and GEO databases using bioinformatic tools. Our study also revealed several favorable relevant immune genes, cells, and pathways in melanoma that could serve as potential therapeutic targets. These findings provide the rationale for further investigation and would aid clinical decision-making in melanoma immunotherapy.

## Data Availability Statement

The datasets analyzed during the present study are available in the TCGA repository (https://portal.gdc.cancer.gov/) and GEO database (https://www.ncbi.nlm.nih.gov/geo/query/acc.cgi?acc=GSE65904).

## Ethics Statement

Ethical review and approval were not required for the study on human participants in accordance with the local legislation and institutional requirements. The patients/participants provided their written informed consent to participate in this study. Ethical review and approval were not required for the animal study because all data are available in online database which have obtained ethical review and approval before. Written informed consent was obtained from the individual(s) for the publication of any potentially identifiable images or data included in this article.

## Author Contributions

CZ and XC were responsible for the literature review and writing *Introduction* and *Discussion* of the manuscript. DD and YW analyzed the bioinformatics data and wrote *Material and Methods* and *Results* sections of the manuscript. All authors contributed to the article and approved the submitted version.

## Funding

The present study was funded by two projects: The Scientific Research Foundation of Jilin Province (nos. 20200601010JC and 20190701061GH).

## Conflict of Interest

The authors declare that the research was conducted in the absence of any commercial or financial relationships that could be construed as a potential conflict of interest.
